# Immune checkpoint inhibitors associated granulomatous small vessel vasculitis accompanied with tubulointerstitial nephritis: a case report

**DOI:** 10.1186/s12882-023-03091-8

**Published:** 2023-03-09

**Authors:** Kenta Tominaga, Kazuhiro Takeuchi, Shoichiro Takakuma, Emi Sakamoto, Saeko Hatanaka, Yusuke Kajimoto, Etsuko Toda, Yasuhiro Terasaki, Shinobu Kunugi, Mika Terasaki, Akira Shimizu

**Affiliations:** 1 Department of Analytic Human Pathology, Nippon Medical School, Tokyo, Japan; 2 Division of Pathology, Nippon Medical School Hospital, Tokyo, Japan

**Keywords:** Immune checkpoint inhibitor, Granulomatous vasculitis, Tubulointerstitial nephritis, Immune-related adverse event, Acute kidney injury

## Abstract

**Background:**

Immune checkpoint inhibitors (ICIs) have provided significant benefits in cancer treatment, but they could develop immune-related adverse events (irAE). ICI-associated renal adverse effects are rare and tubulointerstitial nephritis (TIN) is the most common in the renal irAE. However, only a few case reports of renal vasculitis associated with ICI have been reported. In addition, the characteristics of infiltrating inflammatory cells of ICI-associated TIN and renal vasculitis have been uncertain.

**Case presentation:**

A 65-year-old man received immune checkpoint inhibitors (ICIs), anti-CTLA-4 (cytotoxic T-lymphocyte-associated protein 4) and anti-PD-1 (programmed cell death 1) antibodies for aggravated metastatic malignant melanoma. About 1 week after the second administration of nivolumab and ipilimumab, acute kidney injury developed. A renal biopsy was performed that showed TIN and non-necrotizing granulomatous vasculitis in interlobular arteries. Massive CD3^+^ T cells and CD163^+^ macrophages infiltrated both tubulointerstitium and interlobular arteries. Many infiltrating cells tested positive for Ki-67 and PD-1 ligand (PD-L1), but negative for PD-1. In CD3^+^ T cells, CD8^+^ T cells were predominantly infiltrated, and these cells were positive for Granzyme B (GrB) and cytotoxic granule TIA-1, but negative for CD25, indicating antigen-independent activated CD8^+^ T cells. Infiltration of CD4^+^ T cells was noted without obvious CD4^+^ CD25^+^ regulatory T (Treg) cells. His renal dysfunction recovered within 2 months of treatment with prednisolone in addition to discontinuation of nivolumab and ipilimumab.

**Conclusions:**

We herein reported a case of ICI-related TIN and renal granulomatous vasculitis with infiltration of massive antigen-independent activated CD8^+^ T cells and CD163^+^ macrophages, and none or few CD4^+^ CD25^+^ Treg cells. These infiltrating cells might be a characteristic of the development of renal irAE.

## Background

Immune checkpoint inhibitors (ICIs) have been shown to contribute to the effective treatment of many malignancies including metastatic melanoma [1, 2]. However, these treatments are sometimes associated with active immunity-mediated side effects known as immune-related adverse events (irAE) [3]. IrAE includes dermatologic, gastrointestinal, hepatic, endocrine, and other less common renal adverse effects [4]. In the kidney, tubulointerstitial nephritis (TIN) is the most common pathological feature of renal irAE. In the TIN in renal irAE, T cells are predominant infiltrating cells with infiltration of plasma cells and eosinophils, and its pathologic features are indistinguishable from those of other drug-induced TIN [5]. Furthermore, only a few case reports of renal vasculitis associated with ICI have been reported [6]. In addition, the characteristics of infiltrating inflammatory cells of ICI-associated TIN and renal vasculitis have not been well investigated.

We experienced a case of ICI-associated TIN with renal granulomatous vasculitis and analyzed the characteristics of infiltrating cells in the renal irAE. In our case, dual immune checkpoint pathways, such as programmed cell death 1 (PD-1) / programmed death-ligand 1 (PD-L1) and cytotoxic T-lymphocyte-associated protein 4 (CTLA-4)/B7(CD80/CD86) pathways, were inhibited by ICIs using nivolumab (anti-PD-1 antibody) and ipilimumab (anti-CTLA-4 antibody).

## Case presentation

A 65-year-old man showing acute kidney injury (AKI) was referred to the department of nephrology in our hospital. He has been treated for malignant melanoma. His malignant melanoma under the right first fingernail was diagnosed 2 years ago, and since then, he had shown persistent metastatic lesions at the dorsal side of the right first finger, right wrist, and left lumbar back. Each recurrence was carefully followed-up with extended resection and scheduled treatment using ICIs, nivolumab (anti-PD-1 antibody) 80 mg, and ipilimumab (anti-CTLA-4 antibody) 3 mg/kg intravenously every 3 weeks for a total of 4 doses for aggravated metastatic malignant melanoma. His baseline serum creatinine (Cr) was 0.88 mg/dL and daily oral medication included carvedilol, doxazosin, bezafibrate, lafutidine, and ezetimibe.

About 1 week after the second dose of nivolumab and ipilimumab, AKI developed. His urinary protein excretion was 0.28 g/g urinary Cr. Urinary microscopic examination showed no erythrocytes/high power fields. The laboratory findings were as follows: white blood cells: 4.4 × 10^3^/μL, hemoglobin: 10.8 g/dL, platelets: 168 × 10^3^/μL, serum Cr: 3.51 mg/dL, urea nitrogen: 44.7 mg/dL, total protein/albumin: 5.6/2.5 g/dL, immunoglobulin G (IgG): 895 mg/dL, and IgA/M: 144/47 mg/dL. C-reactive protein level was 23.8 mg/dL, and hypocomplementemia was absent. The anti-neutrophil cytoplasmic antibody (ANCA) titer for myeloperoxidase (MPO) and proteinase 3 (PR3) was within the normal range. Viral antibodies for hepatitis B virus, hepatitis C virus, and human immunodeficiency virus were negative.

**Fig. 1:**
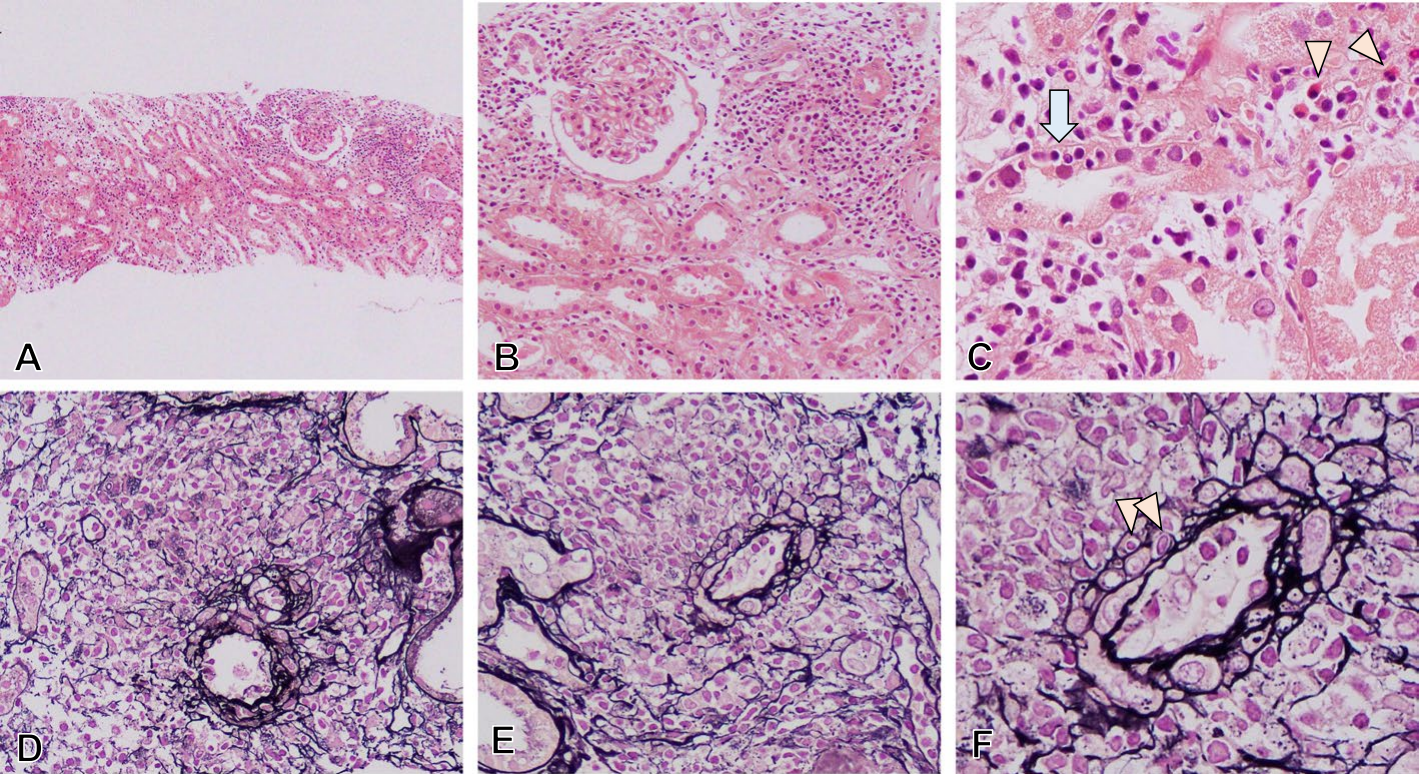
Tubulointerstitial nephritis and granulomatous vasculitis. (**A)** Tubulointerstitial nephritis developed with focal but extensive infiltration of inflammatory cells in the tubulointerstitium. (**B)** Tubulointerstitial nephritis was noted, but glomerular lesions could not be seen. (**C)** Mononuclear cells including eosinophils (arrowhead) infiltrated the interstitium with mild tubulitis (arrow). (**D, E)** Non-necrotizing and granulomatous vasculitis developed in interlobular arteries. (**F)** Infiltration of mononuclear inflammatory cells were found in arterial intima, arterial media (arrowhead), and the arterial adventitia to the perivascular area without fibrinoid necrosis, indicating non-necrotizing and granulomatous arteritis. (**A-C)** HE stain; (**D-F)** PAM stain; (**A)** × 100, (**B)** × 200; (**C, F)** × 1,000; (**D, E)** × 400

**Fig. 2:**
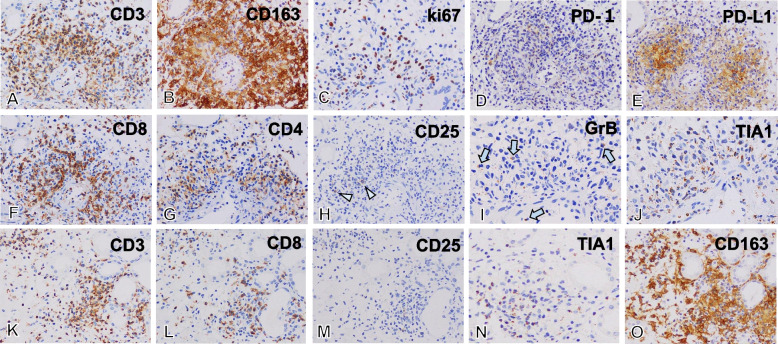
Characteristics of inflammatory cells in the granulomatous arteritis and tubulointerstitial nephritis. In renal granulomatous vasculitis (**A-J**), many CD3^+^ T cells (**A**) and CD163^+^ macrophages (**B**) infiltrated the arterial intima, media, and adventitia up to around the interlobular artery. Many infiltrative inflammatory cells were positive for Ki67 (**C**), indicating that infiltrating cells were proliferating in the vasculitis lesion. (**D, E)** Inflammatory cells were PD-1 negative and PD-L1 positive. (**F, G)** In CD3^+^ cells, abundant CD8^+^ T cells and less predominantly CD4^+^ T cells were noted. (**H)** Only a few CD25^+^ cells (arrowhead) were detected, however almost all cells were negative for CD25. Infiltrating cells were positive for granzyme B (GrB) (arrow in I) and cytotoxic granule T cell intracellular antigen 1 (TIA-1) (**J**), indicating effector and cytotoxic T cells. In tubulointerstitial nephritis (**K-O**), a similar phenotype of infiltrating cells, such as CD3^+^ T cells (**K**), predominantly CD8^+^ T cells (**L**), without CD25 expression (**M**), but positive for TIA-1 (**N**) were noted in interstitium. (**O)** Many CD163^+^ macrophages were also evident in intersitium. (**A-G, K-M, O**) × 200; (**H-J, N)** × 400

On admission, a renal biopsy was performed and showed TIN and renal granulomatous vasculitis in interlobular arteries (Fig. 1). Inflammatory cells infiltrated the interstitium with edema and fibrosis. Necrotizing and crescentic glomerular lesions could not be seen. The infiltrating inflammatory cells were mainly composed of mononuclear cells and a few eosinophils with mild tubulitis. The inflammatory cells had also accumulated within and around interlobular arteries without necrotic lesions, indicating the development of non-necrotizing granulomatous arteritis. Immunofluorescence staining showed no obvious deposition of immunoglobulins and complements in both glomeruli and tubulointerstitium. In the immunohistochemistry for vasculitis lesion (Fig. 2A-J) and TIN lesion (Fig. 2K-O), the infiltrating cells were composed of high numbers of CD3^+^ T cells and CD163^+^ macrophages. Many of these cells had Ki-67 positive nuclei, indicating the proliferation in situ. Inflammatory cells were almost negative for PD-1, while they had a significant expression of PD-L1. Many CD3^+^ T cells demonstrated predominantly CD8^+^ T cells than CD4^+^ T cells, many of which were negative for CD25, but positive for granzyme B (GrB) and cytotoxic granule T cell intracellular antigen 1 (TIA − 1), indicating antigen-independent activated CD8^+^ T cells. CD4^+^ T cells had a slightly fewer distribution than CD8^+^ T cells, and CD25 was negative, indicating no or a small number of CD4^+^ CD25^+^ regulatory T (Treg) cells. In our case, many antigen-independent activated CD8^+^ T cells and CD163^+^ macrophages, and none or few CD4^+^ CD25^+^ Treg cells might be involved in the development of TIN and granulomatous vasculitis in renal irAE.

After the renal biopsy, nivolumab and ipilimumab were stopped and prednisolone 50 mg/day was started as a treatment for ICI-related AKI. Serum Cr levels recovered from 3.51 mg/dL to 1.07 mg/dL within 2 months after treatment, and his renal function was stable thereafter with gradually tapered prednisolone. Nine months after stopping ICI, nivolumab monotherapy 240 mg intravenously was started for metastatic melanoma, and stable renal function continued for 11 months with nivolumab monotherapy.

## Discussion and conclusions

Although ICIs have been proven to greatly contribute to many malignancy treatments including metastatic melanoma [1, 2], excessive immune activation results in the development of irAE [3]. The mechanism of irAE has been uncertain. However, combination therapy using an anti-CTLA-4 antibody and anti-PD-1 antibody appears to be associated with more severe grade toxicities [7].

In the immune system, after elimination by central tolerance mechanisms of the thymus, self-reactive lymphocytes are controlled by peripheral tolerance mechanisms. To maintain peripheral tolerance, both PD-1/PD-L1 and CTLA-4/B7 (CD80/CD86) pathways have a significant role in the inhibition of self-immune responses. Nivolumab is a monoclonal antibody to PD-1 on T cells, which blocks the PD-1/PD-L1 pathway and results in the enhancement of self-inflammatory responses of irAE, including renal irAE [8]. Ipilimumab is a monoclonal antibody to CTLA-4, which blocks the inhibitory signals of CTLA-4/B7 (CD80/CD86) pathway on T cells as well as an exclusive function by Treg cells, and leads to breakdown the inhibitory immune response [9].

Proton pump inhibitor (PPI) is reported as a risk factor for ICI-related AKI [10]. In addition, it has been proposed that ICI may induce PPI and nonsteroidal anti-inflammatory drugs (NSAIDs) related AKI by modifying the T cell immune system which maintains tissue tolerance [11]. In our case, both PPI and NSAIDs were not prescribed throughout his treatment. The inhibition of dual immune-checkpoint pathways, PD-1/PD-L1 as well as CTLA-4/B7 pathways, therefore, might be associated with the enhancement of self-immune responses, which resulted in TIN and renal granulomatous vasculitis.

The current recommendations by major societies including American Society of Clinical Oncology (ASCO), National Comprehensive Cancer Network (NCCN), and Society for Immunotherapy of Cancer (SITC) advocate that ICI treatment should be stopped when the serum Cr is increased three times more than the baseline serum Cr which means Grade 3 of kidney irAE [12]. In our case, therefore, ICIs including nivolumab and ipilimumab were stopped and prednisolone was started, and AKI was recovered within 2 months.

Daxini, et al. [13] reported a review of 20 cases of vasculitis associated with ICI, and ICI-related vasculitis could affect small to large vessels in any organs including the central and peripheral nervous system, uterine and ovarian vessels, retina, peritoneum, and aorta. Giant cell arteritis and granulomatosis with polyangiitis also developed. The pathologic features in each case showed lymphocytic vasculitis with or without systemic symptoms. Especially in small vessel vasculitis, fibrinoid necrosis or/and granulomatous lesion were noted [13–15]. Renal small to medium-sized vasculitis without ANCA is a rare complication of ICI-related kidney injury [16]. In our case, AKI was shown without manifestations of systemic symptoms, indicating renal limited irAE. In addition, TIN as well as non-necrotizing renal granulomatous vasculitis developed in renal irAE.

The pathologic features of immunohistochemical staining in our case revealed massive infiltration of CD3^+^ CD8^+^ T cells in both the interstitium and the interlobular arteries in the renal irAE. Most CD3^+^ CD8^+^ T cells were negative for CD25 and PD-1, but positive for GrB and TIA-1. CD25 is the alpha chain of the trimeric IL-2 receptor and is considered to be the important marker of CD3^+^ T cell activation, including both CD8^+^ T cell and CD4^+^ T cell activation, especially antigen-dependent T cell activation [17]. GrB and TIA-1 are cytotoxic granules in cytotoxic T cells and natural killer cells. These findings indicated that CD8^+^ T cells with CD25^−^ PD-1^−^ GrB^+^ and TIA-1^+^ cells might be characterized by antigen-independent cytotoxic CD8^+^ T cells. In polyarteritis nodosa, known as medium-sized systemic vasculitis, Kobayashi, et al. reported that infiltrating activated CD8^+^ T cells in cutaneous vasculitis represent markers suggesting antigen-independent activation: GrB^+^ CD25^−^ and PD-1^−^ [18]. Tietze, et al. reported that cytokine-based cancer immunotherapy developed antigen-nonspecific activated CD8^+^ T cells which show the same phenotype: GrB^+^ CD25^−^ and PD-1^−^ [19]. In vitro experiments, murine CD8^+^ T cells treated by IL-2 alone (antigen-independent stimulation of T cells) showed a low expression of CD25 and PD-1. These findings support that, in our case, ICIs mediated the expansion of antigen-independent activated CD8^+^ T cells whose phenotype was GrB^+^ CD25^−^ and PD-1^−^, and might induce the TIN and renal vasculitis in renal irAE. In addition to the infiltration of CD8^+^ T cells, CD4^+^ T cells also infiltrated in the TIN and vasculitis lesions. Most CD4^+^ T cells were negative for CD25, indicating that none or few CD4^+^ CD25^+^ Treg cells were noted in TIN and vasculitis lesions.

In our case, massive CD163^+^ macrophages also infiltrated both the vascular area and the interstitium. CD163 is well known as a marker of M2 macrophage. In small vessel vasculitis, urinary soluble CD163 level is known as a significantly better biomarker for active renal vasculitis [20]. In irAE, Tabie, et al. reported a case of acute TIN with infiltration of CD163^+^ M2 macrophages induced by ICI [21]. We considered that CD163^+^ macrophages were involved in the development of ICI-associated TIN and renal granulomatous vasculitis in the present case.

In this report, we experienced a rare renal irAE, TIN and non-necrotizing renal granulomatous vasculitis with infiltration of many antigen-independent activated CD8^+^ T cells, massive CD163^+^ macrophages, and no or a small number of CD4^+^ CD25^+^ Treg cells. These inflammatory cells might be involved in the development of ICI-induced TIN and renal granulomatous vasculitis.

## Data Availability

All data generated or analyzed during this study are included in this published article.

## Abbreviations

ICIs: Immune checkpoint inhibitors
CTLA-4: Cytotoxic T-lymphocyte-associated protein 4)
PD-1: Programmed cell death 1
TIN: Tubulointerstitial nephritis
PD-L1: PD-1 ligand
GrB: Granzyme B
Treg: Regulatory T
AKI: Acute kidney injury
Cr: Creatinine
ANCA: Anti-neutrophil cytoplasmic antibody
MPO: Myeloperoxidase
PR3: Proteinase 3
TIA − 1: Cytotoxic granule T cell intracellular antigen 1

## References

[1] Larkin J, Chiarion-Sileni V, Gonzalez R, Grob JJ, Cowey CL, Lao CD, Schadendorf D, Dummer R, Smylie M, Rutkowski P (2015). Combined Nivolumab and Ipilimumab or monotherapy in untreated melanoma. N Engl J Med.

[2] Wei SC, Duffy CR, Allison JP (2018). Fundamental mechanisms of immune checkpoint blockade therapy. Cancer Discov.

[3] Champiat S, Lambotte O, Barreau E, Belkhir R, Berdelou A, Carbonnel F, Cauquil C, Chanson P, Collins M, Durrbach A (2016). Management of immune checkpoint blockade dysimmune toxicities: a collaborative position paper. Ann Oncol.

[4] Cortazar FB, Marrone KA, Troxell ML, Ralto KM, Hoenig MP, Brahmer JR, Le DT, Lipson EJ, Glezerman IG, Wolchok J (2016). Clinicopathological features of acute kidney injury associated with immune checkpoint inhibitors. Kidney Int.

[5] Cortazar FB, Kibbelaar ZA, Glezerman IG, Abudayyeh A, Mamlouk O, Motwani SS, Murakami N, Herrmann SM, Manohar S, Shirali AC (2020). Clinical features and outcomes of immune checkpoint inhibitor-associated AKI: a multicenter study. J Am Soc Nephrol.

[6] Herrmann SM, Perazella MA (2020). Immune checkpoint inhibitors and immune-related adverse renal events. Kidney Int Rep.

[7] Postow MA, Sidlow R, Hellmann MD (2018). Immune-related adverse events associated with immune checkpoint blockade. N Engl J Med.

[8] Vandiver JW, Singer Z, Harshberger C (2016). Severe hyponatremia and immune nephritis following an initial infusion of Nivolumab. Target Oncol.

[9] Sobhani N, Tardiel-Cyril DR, Davtyan A, Generali D, Roudi R, Li Y (2021). CTLA-4 in regulatory T cells for Cancer immunotherapy. Cancers (Basel).

[10] Seethapathy H, Zhao S, Chute DF, Zubiri L, Oppong Y, Strohbehn I, Cortazar FB, Leaf DE, Mooradian MJ, Villani AC (2019). The incidence, causes, and risk factors of acute kidney injury in patients receiving immune checkpoint inhibitors. Clin J Am Soc Nephrol.

[11] Shirali AC, Perazella MA, Gettinger S (2016). Association of Acute Interstitial Nephritis with Programmed Cell Death 1 inhibitor therapy in lung Cancer patients. Am J Kidney Dis.

[12] Moss EM, Perazella MA (2022). The role of kidney biopsy in immune checkpoint inhibitor nephrotoxicity. Front Med (Lausanne).

[13] Daxini A, Cronin K, Sreih AG (2018). Vasculitis associated with immune checkpoint inhibitors-a systematic review. Clin Rheumatol.

[14] Kao JC, Liao B, Markovic SN, Klein CJ, Naddaf E, Staff NP, Liewluck T, Hammack JE, Sandroni P, Finnes H (2017). Neurological complications associated with anti-programmed death 1 (PD-1) antibodies. JAMA Neurol.

[15] Lemoine M, Dilly B, Curie A, Hebert V, Laurent C, Hanoy M, Grange S, Guerrot D, Francois A, Bertrand D (2019). Ipilimumab-induced renal granulomatous arteritis: a case report. BMC Nephrol.

[16] Gallan AJ, Alexander E, Reid P, Kutuby F, Chang A, Henriksen KJ (2019). Renal Vasculitis and Pauci-immune glomerulonephritis associated with immune checkpoint inhibitors. Am J Kidney Dis.

[17] Kim HP, Imbert J, Leonard WJ (2006). Both integrated and differential regulation of components of the IL-2/IL-2 receptor system. Cytokine Growth Factor Rev.

[18] Kobayashi M, Ogawa E, Okuyama R, Kanno H (2018). In vasculitis of small muscular arteries, activation of vessel-infiltrating CD8 T cells seems to be antigen-independent. Virchows Arch.

[19] Tietze JK, Wilkins DE, Sckisel GD, Bouchlaka MN, Alderson KL, Weiss JM, Ames E, Bruhn KW, Craft N, Wiltrout RH (2012). Delineation of antigen-specific and antigen-nonspecific CD8(+) memory T-cell responses after cytokine-based cancer immunotherapy. Blood.

[20] O'Reilly VP, Wong L, Kennedy C, Elliot LA, O'Meachair S, Coughlan AM, O'Brien EC, Ryan MM, Sandoval D, Connolly E (2016). Urinary soluble CD163 in active renal Vasculitis. J Am Soc Nephrol.

[21] Tabei A, Watanabe M, Ikeuchi H, Nakasatomi M, Sakairi T, Kaneko Y, Maeshima A, Kaira K, Hirato J, Nojima Y (2018). The analysis of renal infiltrating cells in acute Tubulointerstitial nephritis induced by anti-PD-1 antibodies: a case report and review of the literature. Intern Med.

